# Comparison of antimicrobial and wound-healing effects of silver nanoparticle and chlorhexidine mouthwashes: an in vivo study in rabbits

**DOI:** 10.1007/s10266-022-00690-z

**Published:** 2022-02-26

**Authors:** Amirhossein Moaddabi, Parisa Soltani, Carlo Rengo, Sahar Molaei, Seyed Jaber Mousavi, Mojdeh Mehdizadeh, Gianrico Spagnuolo

**Affiliations:** 1 Department of Oral and Maxillofacial Surgery, Dental Research Center, Mazandaran University of Medical Sciences, Sari, Iran; 2 Faculty of Dentistry, Mazandaran University of Medical Sciences, Sari, Iran; 3 Department of Oral and Maxillofacial Radiology, Dental Implants Research Center, Dental Research Institute, School of Dentistry, Isfahan University of Medical Sciences, Isfahan, Iran; 4 Department of Neurosciences, Reproductive and Odontostomatological Sciences, University of Naples “Federico II”, Naples, Italy; 5 Students’ Research Committee, Faculty of Dentistry, Mazandaran University of Medical Sciences, Sari, Iran; 6 Department of Community Medicine, Faculty of Medicine, Mazandaran University of Medical Sciences, Sari, Iran

**Keywords:** Mouthwash, Oral surgery, Chlorhexidine, Silver nanoparticle, Antimicrobial, Wound healing

## Abstract

The objective is to formulate a silver nanoparticle mouthwash and then evaluate its antimicrobial and wound-healing effects in rabbit animal models. Microbial samples were collected from the oral cavity of 60 rabbits. Thereafter, standardized wounds were created in the lateral border of the tongue on the right side for all rabbits. After surgery, digital photographs were obtained from the wounds with standardized settings. To characterize the silver nanoparticles used in the synthetic mouthwash, transmission electron microscopy (TEM) and digital light scattering analysis were used. The animal models were then randomly divided into 4 groups: group 1 received 9.80 wt% silver nanoparticle mouthwash; group 2 received all the ingredients of the formulated mouthwash except for silver nanoparticles; group 3 received chlorhexidine 2.0% mouthwash; and the negative control group did not receive any postoperative mouthwash. Microbial samples were collected from oral cavity of the rabbits each day for four postoperative days. Colony-forming unit (CFU) counts were compared post-operatively with the pre-operative counts. In addition, standardized digital photographs were taken each day from the wounds and the area of the wounds was compared in postoperative and pre-operative images. Data were statistically analyzed using one-way ANOVA and repeated measures variance analysis (*α* = 0.05). TEM revealed spherical morphology of silver nanoparticles and digital light scattering showed an average size of 5 nm and optimal distribution of the nanoparticles. CFU count significantly decreased in groups 1 and 3 (*P* < 0.001), while it significantly increased in groups 2 and 4 (*P* < 0.001). Moreover, a significant difference was observed between the experimental groups (*P* < 0.001). In addition, wound area decreased significantly in all groups (*P* < 0.001). However, the difference between wound areas in the groups was not significant, except for the 4th postoperative day (*P* < 0.001). However, the antibacterial effects and the wound-healing characteristics of the synthetic silver nanoparticle and chlorhexidine mouthwashes were not significantly different (*P* > 0.05). Silver nanoparticle mouthwash possesses favorable antibacterial and wound-healing effects. The formulated 9.80 wt% silver nanoparticle mouthwash with a particle size of 5 nm can be a promising alternative for application after oral surgical procedures.

## Introduction

Several methods are used for control of dental plaque including mechanical and chemical techniques. In patients who have recently experienced trauma or oral surgical procedures, adequate mechanical plaque control is not possible. In these cases, chemical methods for plaque control, such as mouthwashes can be used [1, 2]. Antimicrobial mouthwashes can decrease the bacterial load in the oral cavity, and therefore, help in alleviating various oral diseases and inflammatory conditions [3, 4]. Chlorhexidine is the routine antimicrobial mouthwash in dental procedures [5]. One of the advantages of chlorhexidine is its ability to adhere to oral tissues, allowing it to release slowly and provide antimicrobial effects over a certain period of time. The mechanism of action of chlorhexidine is adherence to the bacterial cell wall [6]. Due to its property of substantivity, chlorhexidine adsorbs to tooth surfaces destabilizes bacterial cell walls and interferes with cellular osmosis, leading to lysis of bacterial cells. [7]. In lower concentrations, chlorhexidine is bacteriostatic, while higher concentrations of chlorhexidine are bactericidal [8]. Long-term use of chlorhexidine can result in discoloration of teeth, restorations, oral mucosa, and tongue dorsum. In addition, bad taste, xerostomia, and detrimental effects of oral microflora is other disadvantages of chlorhexidine [6]. Therefore, researchers have sought other antimicrobial agents for using as mouthwash [6, 9–11].

Silver-based antimicrobial agents have been recently developed for application in infections and wound healing [12]. Among metals with antimicrobial characteristics, silver possesses potent antibacterial effects. In addition, silver is nontoxic and non-mutagenic for human cells [13]. Nanoparticles of silver are introduced as potential agents for using in mouthwashes [14, 15]. Nanoparticles provide a larger contact surface and thus, increase the antimicrobial effects of silver [16]. Silver nanoparticles demonstrate antimicrobial effects against a variety of Gram-negative, Gram-positive, aerobic, anaerobic, and even vancomycin-resistant species [17–19]. In addition, silver nanoparticles have shown significant wound-healing effects. Studies have demonstrated a dose-dependent effect for silver nanoparticles in accelerating wound healing and preventing scar formation, leading to enhanced esthetic results as well as hair eruption [20]. Animal studies have shown that therapeutic agents containing silver nanoparticles are effective in preventing pus formation in wounds, improving collagen alignment, and subsequently decreasing scar formation [21–23]

A few studies have been carried out to evaluate the effects of silver nanoparticles in the oral environment. Most of these studies are in vitro studies evaluating the antimicrobial effects of nano-silver on oral microflora [24–26]. Therefore, in this study, we aimed to first develop a silver nanoparticle mouthwash and then evaluate its antimicrobial and wound-healing effects in rabbit animal models.

## Materials and methods

The present study was approved by the Ethical Committee of Mazandaran University of Medical Sciences (#IR.MAZUMS:REC.1397.1588). The sample size in each group was calculated as 12 rabbits considering *α* = 0.05 and *β* = 0.2. Due to a chance for premature loss of animal models, a sample size of 15 was considered for each group. The selected models were male New Zealand rabbits, being mature and young, with an average weight of 2.5 kg.

**Fig. 1:**
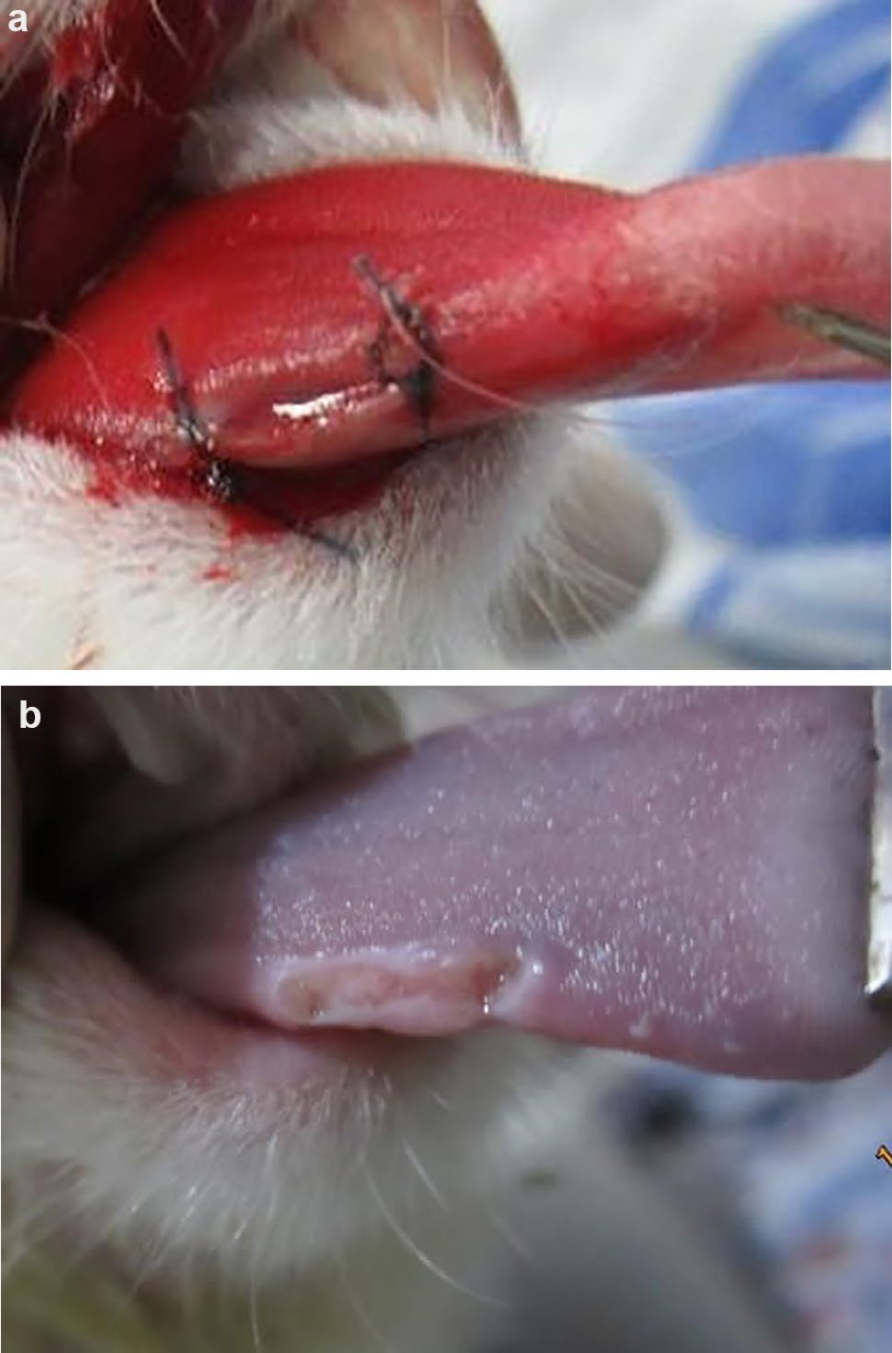
Photograph of the incision on the right lateral border of the tongue in one of the animal models **a** right after the operation and **b** 4 days after the operation

First, saliva samples were collected from rabbits using swabbing technique and transferred to Muller Hinton agar media for culturing. The plates were kept for 24 h in 20 °C temperature and then the colony forming units (CFUs) were counted. The next day, the rabbits were sedated by intramuscular injection of 60 units of ketamine 10% and 40 units of Xylazine 2% using a 1 mL insulin syringe. After sedation, Xyla-P topical cream (Tehran Chemie, Tehran, Iran) was used for local anesthesia. Thereafter, a 1-cm-long, 1-mm deep wound was created in the right lateral border of the tongue of all rabbits using #12 carbon steel bistoury blade (Technocut, HMD, Faridabad, India). To control bleeding, the ends of the wound were sutured using #4-0 monofilament polyamide nylon suture with a reverse cutting 19-mm 3/8 needle (Supa, Tehran, Iran) (Fig. 1).

The rabbits were then divided into four groups using a random numbers chart; group 1: synthetic mouthwash with silver nanoparticles; group 2: synthetic mouthwash without silver nanoparticles; group 3: control group using chlorhexidine mouthwash; and group 4: negative control group without administering mouthwash.

For group 1, a synthetic mouthwash containing 80.38 wt% water, 9.80 wt% glycerin, 9.80 wt% nano-silver suspension with 400 ppm concentration, and 0.02 wt% sodium saccharine was used. The silver nanoparticles were characterized using transmission electron microscopy (TEM, CM30, Philips, Amsterdam, The Netherlands). In addition, silver nanoparticles were characterized using sonicator probe for 10 min with Am = 20, to demonstrate the sustainability of the nanoparticle suspension and determine the size and distribution of nanoparticles. For group 2, all other components of the synthetic mouthwash except silver nanoparticles were mixed and used. For group 3, alcohol-free chlorhexidine 0.2% solution (Shahre Daru, Tehran, Iran) was used. Four drops of each mouthwash were administered every 8 hours for 4 days using a small syringe. The animals in group 4 did not receive any mouthwash. Microbial samples were collected from all animal models each day for 4 days. CFUs were counted and compared with the pre-operative values to evaluate the antimicrobial activity of the mouthwashes. To assess the wound-healing effects of the mouthwashes, standardized digital photographs were taken right after surgery with a distance of 8 cm from the wounds, without zooming using a digital camera (IXUS 115 H5, Canon, Tokyo, Japan). The rabbits were fixed using a restrainer for the photographic sessions. Photographic images were obtained each day for 4 days after administration of the mouthwash for the third time that day. The area of the wounds was calculated using Digimizer (https://www.digimizer.com/) after calibration of the digital camera. The area of wounds was compared post-operatively with the pre-operative values.

Mean and standard deviation values were used to present quantitative variables. One-way ANOVA and repeated measures variance analysis were used for statistical analysis (*α* = 0.05) by Statistical Package for the Social Sciences (SPSS, v.16, IBM, IL, USA).

## Results

**Fig. 2:**
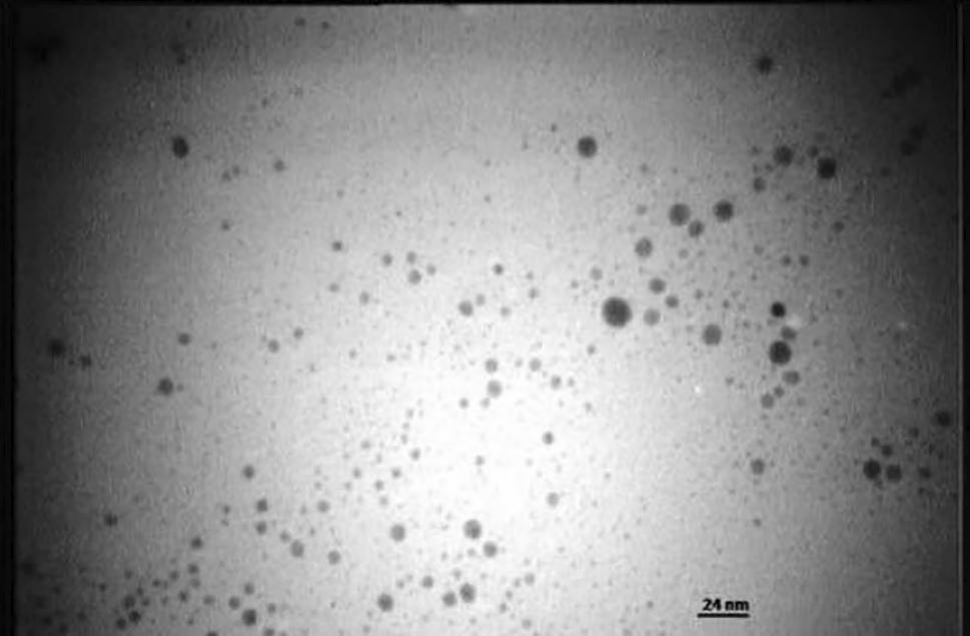
Transmission electron microscopy (TEM) image of silver nanoparticles

**Fig. 3:**
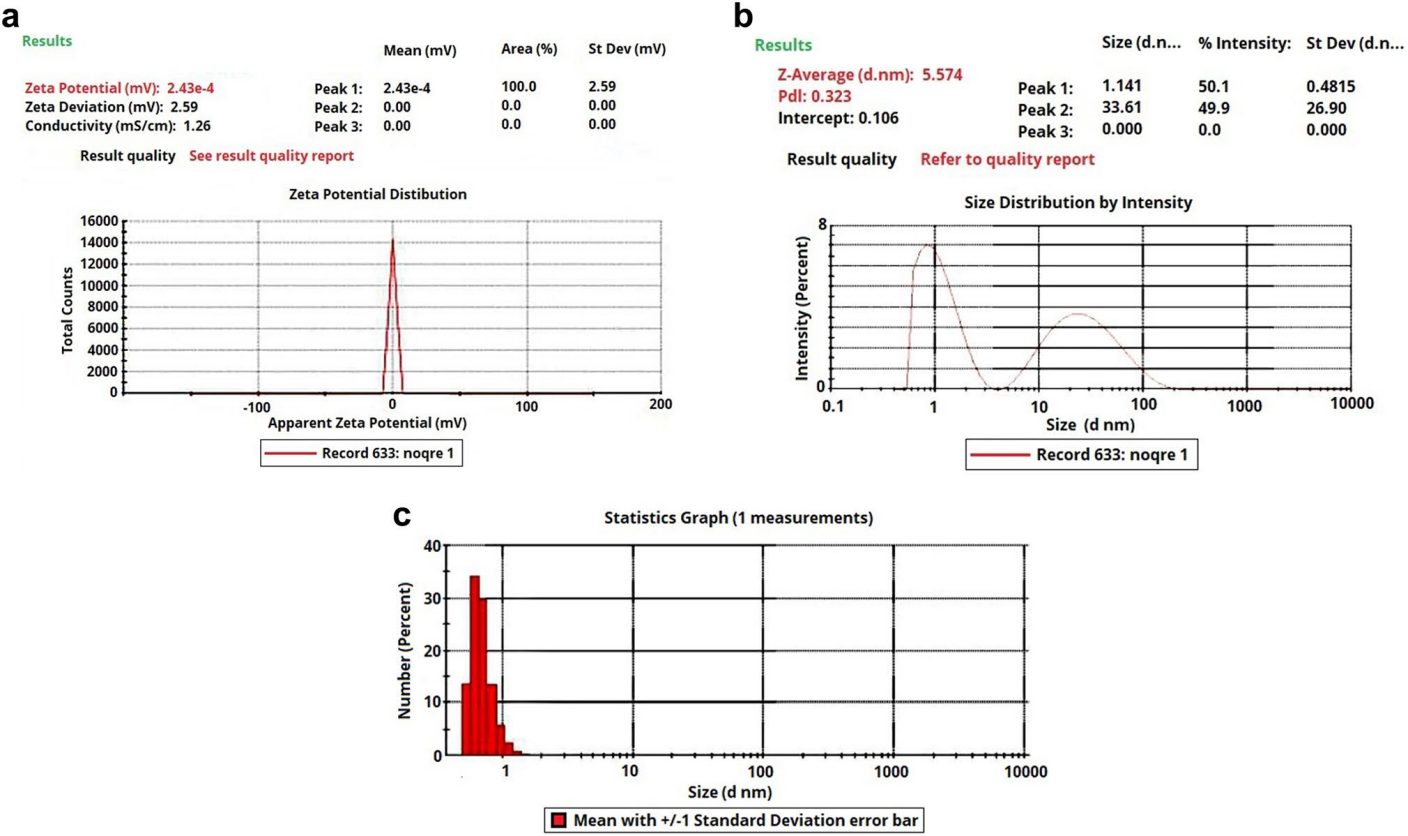
Sonication results of silver nanoparticle suspension: **a** zeta potential demonstrating the sustainability of the suspension, **b** distribution of silver nanoparticles, and **c** number of silver nanoparticles with different sizes

Based on TEM, silver nanoparticles were spherical and mostly homogenous in size (Fig. 2). In addition, dynamic light scattering analysis revealed a low zeta potential (Fig. 3a). However, since other solvents were used combined with water, the mouthwash suspension was sustainable. In addition, the Z-average of silver nanoparticles was 5.57 nm (Fig. 3b) and the polydispersity index was 0.3 revealing appropriate homogeneity in size of silver nanoparticles (Fig. 3c).

The mean values of CFU counts and wound areas in different groups are presented in Tables 1 and 2, respectively. No significant difference was observed among the groups in surgery day CFU counts and wound area (*P* = 0.053 and *P* = 0.473, respectively).

**Table 1:** Mean value of CFU counts in the groups in different days

	Mean (SD)
Group 1	
Surgery day	47.50 (2.12)
Day 1	30.43 (1.41)
Day 2	25.50 (9.19)
Day 3	10.98 (1.41)
Day 4	5.34 (1.01)
*P* value	< 0.001
Group 2	
Surgery day	50.50 (2.72)
Day 1	54.58 (3.54)
Day 2	70.00 (14.14)
Day 3	93.63 (12.73)
Day 4	108.76 (21.66)
*P* value	< 0.001
Group 3	
Surgery day	46.67 (2.09)
Day 1	31.00 (6.82)
Day 2	29.67 (18.93)
Day 3	10.67 (15.28)
Day 4	6.67 (2.93)
*P* value	< 0.001
Group 4	
Surgery day	49.00 (6.83)
Day 1	48.76 (5.66)
Day 2	62.02 (9.90)
Day 3	80.00 (28.28)
Day 4	101.40 (12.52)
*P* value	< 0.001

**Table 2:** Mean value of wound area in the groups in different days

	Mean (SD)
Group 1	
Surgery day	10.37 (0.86)
Day 1	10.29 (0.56)
Day 2	9.42 (0.85)
Day 3	8.93 (0.92)
Day 4	7.72 (0.81)
*P* value	< 0.001
Group 2	
Surgery day	10.01 ± 0.75
Day 1	10.01 ± 0.42
Day 2	9.87 ± 0.74
Day 3	9.10 ± 0.75
Day 4	8.20 ± 0.74
*P* value	< 0.001
Group 3	
Surgery day	10.38 ± 0.62
Day 1	10.33 ± 0.35
Day 2	9.74 ± 0.61
Day 3	9.22 ± 0.62
Day 4	8.19 ± 0.63
*P* value	< 0.001
Group 4	
Surgery day	10.16 (0.75)
Day 1	10.16 (0.66)
Day 2	9.63 (0.78)
Day 3	9.32 (0.83)
Day 4	8.99 (0.80)
*P* value	< 0.001

During the postoperative days, CFU counts significantly decreased in groups 1 and 3 (*P* < 0.001). However, in groups 2 and 4, bacterial load significantly increased during the postoperative days (*P* < 0.001). The groups showed statistically significant difference in CFU counts (*P* < 0.001). However, no significant difference was observed between groups 1 and 3 (*P* > 0.05).

In addition, wound area decreased significantly in all groups (*P* < 0.001). However, the difference between wound areas in the groups was not statistically significant, except for the 4th postoperative day (*P* < 0.001). Comparing groups 1 and 3, no significant difference was observed in the wound-healing effects of the synthetic silver nanoparticle and chlorhexidine mouthwashes (*P* > 0.05).

## Discussion

In the present study, the 9.8 wt% silver nanoparticle mouthwash significantly improved wound healing after incision in the lateral border of the tongue of rabbits. In addition, application of silver nanoparticle mouthwash consistently decreased the CFU counts in the oral cavity of the animal models.

Control of dental plaque formation is an important step in preventing periodontal diseases, dental caries, and other disorders, which is usually done using mechanical and chemical methods [27, 28]. Chemical methods are particularly used when appropriate mechanical control is not possible due to conditions such as surgery or mental and physical disabilities. One of the most convenient methods of chemical plaque control is using mouthwash. Chlorhexidine is considered as a standard agent for chemical plaque control. It is effective against oral bacteria such as *Streptococcus mutans* [19]. However, its long-term use can have several undesirable side effects. Therefore, researchers have explored other alternative agents to be used as antibacterial mouthwashes.

The antimicrobial activity of silver nanoparticles is largely dependent on their sizes [29, 30]. The maximum biocidal activity belongs to nanoparticles smaller than 10 nm, as they are small enough to penetrate the bacterial cells [17, 31]. Therefore, in this study silver nanoparticles smaller than 10 nm were used in the synthetic mouthwash. In addition, the antibacterial effects of silver-based agents increase in higher concentrations [32]. However, higher concentrations of silver can lead to unfavorable taste and potential toxicity of the mouthwash. Studies have shown that although silver nanoparticles can accumulate in the liver, kidneys, testes, lungs, blood, and brain, these particles are not toxic in doses taken for dental purposes [33]. Nevertheless, further studies on potential toxicity of nano-silver-based solutions with different concentrations and different particle sizes are needed [30].

In a study by Sadeghi et al., it was reported that the solution containing silver nanoparticles was comparable with chlorhexidine in its activity against *Streptococcus sanguis* and *Actinomyces viscous*, which are two of the bacterial species present in the oral cavity [24]. Furthermore, Besinis et al. concluded that silver nanoparticles are more potent antimicrobial agents compared to chlorhexidine [26]. A study performed by Esfahanian et al. [25] showed that chlorhexidine mouthwash has superior antibacterial activity compared with nano-silver solution for aerobic and anaerobic bacteria. In the present study, chlorhexidine and silver nanoparticle mouthwash showed statistically similar antimicrobial effects.

Wound healing is another important characteristic for mouthwashes administered after oral surgery. Silver nanoparticles have shown promising results in healing of burn wounds, diabetic ulcers, and other skin wounds [34–37]. However, limited studies have examined the effects of silver nanoparticles on wound healing in the oral cavity. Prasetyo et al. reported that addition of silver nanoparticles to CoePaK^®^ enhanced its wound-healing properties after excision of the palatal tissue in rat models [38]. Another in vivo study in rabbits performed by Ghanbari et al. showed positive potential effects of a periodontal dressing containing silver nanoparticles on wounds after gingivectomy [39]. The present study revealed significant wound-healing effects of silver nanoparticles which can be attributed to the efficient anti-inflammatory properties of silver. Silver nanoparticles reduce cytokine release and decrease lymphocyte and mast cell infiltration, thereby inhibiting further inflammatory responses and contributing in propagation of wound healing [37, 40, 41].

One limitation for the present study was that formulating mouthwashes with different concentrations of silver nanoparticles was not possible due to financial constraints. Nevertheless, our findings can be useful for future studies investigating different properties of silver nanoparticle mouthwashes.

## Conclusion

Application of silver nanoparticle mouthwash after creating an incision on the tongues of rabbit models significantly reduced the number of bacteria in the oral cavity. Furthermore, silver nanoparticle mouthwash had significant positive effects on postoperative wound healing. The formulated mouthwash with a particle size of 5 nm and silver nanoparticles wt. percentage of 9.80% can be a promising alternative for application after oral surgical procedures.
